# Laparoscopic adrenal surgery: ten-year experience in a single institution

**DOI:** 10.1186/1471-2482-13-S2-S5

**Published:** 2013-10-08

**Authors:** Giovanni Conzo, Daniela Pasquali, Cristina Della Pietra, Salvatore Napolitano, Daniela Esposito, Sergio Iorio, Annamaria De Bellis, Giovanni Docimo, Fausto Ferraro, Luigi Santini, Antonio Agostino Sinisi

**Affiliations:** 1 Department of Anesthesiologic, Surgical and Emergency Science VII Division of General and Endocrine Surgery, Second University of Naples - Italy; 2 Department of Cardio-Thoracic and Respiratory Science Endocrinology Unit, Second University of Naples - Italy

## Abstract

**Background:**

Minimal invasive adrenalectomy has become the procedure of choice to treat adrenal tumors with a benign appearance, ≤ 6 cm in diameter and weighing < 100 g. Authors evaluated medium- and long-term outcomes of laparoscopic adrenalectomy (LA), performed for ten years in a single endocrine surgery unit.

**Methods:**

We retrospectively reviewed 88 consecutive patients undergone LA for lesions of adrenal glands from 2003 to 2013. The first 30 operations were considered part of the learning curve. Doxazosin was preoperatively administered in case of pheochromocytoma (PCC), while spironolactone and potassium were employed to treat Conn's disease. Perioperative cardiovascular status modifications and surgical and medium- and long-term results were analyzed.

**Results:**

Forty nine (55.68%) functioning tumors, and one (1.13%) bilateral adrenal disease were identified. In 2 patients (2.27%) a supposed adrenal metastasis was postoperatively confirmed, while in no patients a diagnosis of incidental primitive malignancy was performed. There was no mortality or major post operative complication. The mean operative time was higher during the learning curve. Conversion and morbidity rates were respectively 1.13% and 5.7%. Intraoperative hypertensive crises (≥180/90 mmHg) were observed in 23.5% (4/17) of PCC patients and were treated pharmacologically with no aftermath. There was no influence of age, size and operative time on the occurrence of PCC intraoperative hypertensive episodes. Surgery determined a normalization of the endocrine profile. One single PCC persistence was observed, while in a Conn's patient, just undergone right LA, a left sparing adrenalectomy was performed for a contralateral metachronous aldosteronoma.

**Conclusions:**

LA, a safe, effective and well tolerated procedure for the treatment of adrenal neoplasms ≤ 6 cm, is feasible for larger lesions, with a similar low morbidity rate. Operative time has improved along with the increase of the experience and of the technological development. Preoperative adrenergic blockade did not prevent PCC intraoperative hypertensive crises, but facilitated the control of the hemodynamic stability.

## Introduction

Minimally invasive adrenalectomy (MA), thanks to a lower morbidity rate, a shorter hospital stay, and a more rapid recovery than "open" surgery, increasing patients satisfaction and comfort, has become the standard of care for the management of functioning and non-functioning adrenal neoplasms with a benign appearance, ≤ 6 cm in diameter and weighing < 100 g. In those cases without signs of locoregional infiltration or malignancy, laparoscopic adrenalectomy (LA) has been proposed even for tumors larger than 6 cm in diameter, but it may be associat with longer operative times, a greater blood loss, a higher conversion rate, and further several complications [1-5]. Moreover, a higher risk of malignancy and of capsular disruption with seeding is advocated. Currently indication to LA for lesions > 6 cm is still a matter of debate and experienced endocrine surgeons are divided between supporters [3,5-7] and detractors [8,9]. Nevertheless it has been shown that MA, allowing earlier mobilization, is associated with lower rates of pulmonary infections, thromboembolic complications and mortality than traditional surgery [10]. Progress in the fields of anesthesiology and surgery, a better understanding of the pathophysiology of the adrenal diseases, and an appropriate preoperative blood pressure control contributed to obtain better outcomes. A reduced manipulation of the adrenal glands, displacing the surrounding structures without "handling" the tumor, primary vascular control whenever possible, and sparing of the adrenal capsule, avoiding its rupture and tumor spillage, are the treatment guidelines [3,4,7,10-12].

Authors report the results of a retrospective audit of 88 patients with adrenal lesions who had undergone LA in the last ten years, in a referral endocrine university surgery unit. We evaluated and compared our indications and outcomes with those of the published series.

### Materials and methods

#### Study design

Review of all recorded clinical data was performed for patients who underwent LA for adrenal neoplasm, between January 2003 and January 2013. An asymptomatic lesion, incidentally discovered, and not associated with a predominant hormonal secretion, was considered as an "incidentaloma". Surgical indications included PCCs up to 8 cm in diameter, incidentalomas up to 6 cm, myelolipomas up to 12 cm, adrenal metastases < 6 cm, in patients with an ASA risk not over level III, and age less than 80 years. Suspected primary malignant adrenal tumor was considered as a contraindication to LA. Outcomes measure for our analysis were 30-day mortality and complication rate, and functional medium- and long-term results. All surgical procedures were performed by an experienced endocrine and laparoscopic surgeon, associated with a collaborative team, performing together a LA repetitively. In most cases, patients were referred to surgery by regional endocrinology units, following a comprehensive endocrinological assessment. Data about patients, adrenal pathology, surgery, and intraoperative blood pressure (BP) levels were retrospectively collected. Diagnostic imaging consisted of ultrasonography, computed tomography (CT), magnetic resonance imaging, metaiodobenzylguanidine scintigraphy in all PCC patients, in whom a genetic study looking for mutations of the RET proto-oncogene was performed as well. Anesthesia chart and pathology report of each patient were obtained. Systolic blood pressure (SBP) levels ≥ 180 mmHg were considered hypertensive crises, and levels < 90 mmHg were considered hypotensive crises. Influence of age, tumor size and operative time on occurrence of PCC intraoperative hypertensive episodes were also evaluated. 88 patients, who underwent adrenalectomy, were included in the study (Table1).

According to Zhu [11], until BP and HR stabilization was achieved, (BP < 160/90 mm Hg for at least 24 hours before surgery and HR< 100 beats min), in absence of electrocardiogram ST-T changes for at least 1 week, every PCC patient received a preoperative pharmacological therapy consisting of an alpha1 blocker - doxazosin- at least 15 days before surgery, with a mean dose of 6 mg daily (range 2-10) and a mean therapy duration of 23 days (range 14-52). A beta blocker (atenolol, 50 mg daily) was added in 2 cases with tachycardia (11.7%). A calcium antagonist (amlodipine besylate, 5 mg daily) in 1 case (5.88%), and an angiotensin receptor blocker (valsartan, 160 mg daily) in 1 case (5.88 %) represented the associated previous drug therapy. In each PCC case plasma volume was preoperatively expanded using crystalloid solutions. Postoperative hypotensive crises were treated by hydrocortisone and crystalloids. In Conn's disease patients, with low serum levels of potassium, a preoperative therapy with potassium and spironolactone was indicated. Postoperatively, patients were not routinely admitted to the Intensive Care Unit. In no patient peridural analgesia was employed. Patients were discharged if they had no cardiovascular complains or pain, and had begun oral feeding. In PCC patients follow-up consisted of 6 month, then yearly testing of urinary metanephrine levels and abdominopelvic CT scans with contrast agent.

#### Anesthesia

Patients received general anesthesia without epidural anesthesia. All operations were performed using orotracheal intubation, without local anaesthesia of the upper airways. Invasive arterial pressure monitoring was routinely used for PCC patients. A central venous catheter was selectively previously placed. No pulmonary catheters were used. Hemodynamic data were recorded. Heart rate, systolic and diastolic blood pressure, central venous pressure were recorded before anesthesia induction, after CO_2 _inflation, before and after adrenalectomy. After induction of anesthesia, with remifentanil (0.25 mcg/kg/min) and propofol (2 mg/kg), cisatracurium besylate (0.2 mg/kg ), which was also used as a muscle relaxant during surgery, was administered. Anesthesia was maintained with inhalation of sevoflurane 1% and oxygen 50%, supplemented with remifentanil infusion. Muscle relaxation during the operation was maintained with intermittent boluses under neuromuscular monitoring guide. Blood loss and infused fluid volume during surgery were also recorded. Intraoperative treatment of PCC hypertensive crises consisted of intravenous administration of nitroprusside (initial dose: 0.2 mcg/kg/min, administered by continuous intravenous infusion; maintenance dose was titrated upward to a maximum of 10 mcg/kg/min), esmolol (1 mg/kg bolus dose over 30 seconds followed by a 150 mcg/kg/min infusion, if necessary, or a loading dosage infusion of 500 mcg/kg/min for one minute followed by a four-minute maintenance infusion of 50 mcg/kg/min, adjusting the infusion rate as required up to 300 mcg/kg/min to maintain desired HR and/or BP), urapidil (starting dose: 0.25-0.4 mg/kg or 25 mg; maintenance dose: 9 mg/h of continuous intravenous infusion), clonidine (single bolus: 75-150 µg in 5 min, or by continuous infusion: 0.4-5 µg/min).

#### Surgery

Operative time was considered as the period from skin incision to wound dressing. An antibiotic and antithrombotic prophylactic therapy was administered in all cases. 89 adrenalalectomies were performed using a standard transperitoneal lateral laparoscopic approach, with 4 trocars if the tumor was in the right adrenal gland, and usually 3 trocars if it was in the left one. The patients were placed in the left or right lateral decubitus position (on the side opposite to the tumor), of about 30° for right LA and of about 90° for left LA. In each case pneumoperitoneum was induced by Hasson trocar, according to an "open" technique, and was maintained at 12-14 mmHg with carbon dioxide (CO_2_). Harmonic scalpel™ (Ethicon Endo Surgery INC - Johnson & Johnson, NJ, USA) or LigaSure™vessel sealing system (Tyco, Boulder CO, USA) were routinely employed for adrenal glands dissection. A bipolar coagulation was routinely preferred for hemostasis whenever necessary. In the surgical intervention, the first step in most cases was the vascular control of the main adrenal vein by clips. In two cases a linear stapler was utilized. Whenever necessary, the lateral and posterior connections of the right hepatic lobe were incised and the liver was superiorly and medially retracted, taking particular care in Cushing's patients, in whom the liver may be very fragile. In case of a right tumour larger than 6 cm, the first step was mobilization of the adrenal gland on both the upper and medial sides, in order to have a safer access to the inferior vena cava and the ipsilateral renal vein. During a left LA, a wide left colon mobilization is routinely carried out. The surgical specimens were extracted in retrieval bags through a mini-laparotomy at the site of one of the trocars. A off-suction 20 Fr drain was routinely placed and removed after about 1-2 days. Full mobilization and free eating were recommended on the first postoperative day.

## Statistics

Data were expressed as mean, unless otherwise specified. Statistical analysis was performed with SPSS version 11.5 (SPSS^©^, Chicago, IL, USA). Significance was assigned with a p value <0.05.

## Results

### 

#### Demographics

68 women and 22 men, with a mean age of 48 years (range 22-74) were enrolled. A right adrenal gland tumor in 50 cases (56.8%) and 1 bilateral tumor was reported. Associated pathologies included hypertension in 53 patients (60.2%), insulin-dependent diabetes in 19 patients (21.6%) and dilated cardiomyopathy in 7 patients (7.95%). There was one case of genetically determined polyendocrine syndromes: a multiendocrine neoplasia (MEN) 2A, due to a triple mutation of the RET proto-oncogene (634,640,700).

Tumor sizes are reported in table 1. In each patient, surgery determined a normalization of the pathological hormonal serum levels. Only one PCC patient, affected by persisting disease, due to retrocaval residual adrenal tissue, showed a persistent postoperative elevation of metanephrine levels. Functioning tumors were present in 49 cases (55.68%). 17 patients (19.3%) were affected by PCC, 16 (18.18%) by Conn's disease, 16 (18.18%) by Cushing's disease, 1 (1.13%) by a voluminous myelolipoma, while in 34 cases (38.63%) an incidentaloma was present. In 2 cases (2.72%), adrenal cysts were reported by the definitive pathology. Two metastases (one in the right and one in the left adrenal gland) appeared respectively 13 and 3 years after the surgical treatment of a breast and a renal cancer. Tumor mean size was of 5.86 cm (range 1.1-12). Aldosteronomas were associated with the smallest size (1.1 cm), while myelolipoma was the largest observed neoplasm (12 cm). In PCC patients preoperative mean 24-hour urinary catecholamines concentration was 457.23 ± 215.37 pg/dl (normal value 0-115 pg/dl), and doxazosin was well tolerated without important side effects, allowing an efficacious BP and HR control. Testing for RET mutations led to identification of a triple mutation (634, 640, 700) in the patient suffering from MEN 2A [13]. No other tested patients were found to have mutations of this proto-oncogene.

**Table 1:** Demographics

		%
**Mean age (years)**	48	

**Male patients**	22	25

**Site: right**	50	56.8

** Left**	39	44.2

**Mean size (cm)**	5.86	

**ASA score 1-2**	74	84

** 3**	14	16

**PCC**	17	19.3

**Aldosteronoma**	16	18.2

**Cushing's disease**	16	18.2

**Myelolipoma**	1	1.1

**Incidentaloma**	34	38.6

**Adrenal metastasis**	2	2.2

**Cyst**	2	2.2

PCC: Pheochromocytoma

#### Surgery

Twenty two (25%) patients had undergone previous abdominal surgery, with a laparoscopic or laparotomic approach. A left LA was performed for a PCC relapse and for a renal cell carcinoma adrenal metastasis in two patients, respectively previously undergone traditional left adrenalectomy (PCC) and left nefrectomy (renal cell carcinoma). No simultaneous laparoscopic procedures were performed. Overall mean operative time was 137.33 minutes (range 100-180) during the learning curve and 130.94 minutes (range 90-180) after this time, with a statistically significant difference; mean blood loss was 149.7 ml (range 50-280) (Table 2). No patient required intra or postoperative blood transfusion. Harmonic scalpel™(Ethicon Endo Surgery INC - Johnson & Johnson, NJ, USA) or LigaSure™vessel sealing system (Tyco, Boulder, CO, USA) showed a similar efficacy in tissue dissection and hemostasis control. There was no mortality. A part from PCC hyperthensive crises, the most significant observed intraoperative complications were an injury of the inferior vena cava, sutured and treated by Floseal^® ^Hemostatic Matrix (Baxter Zurich- Switzerland) and oxidized cellulose (Tabotamp Fibrillar Johnson & Johnson, NJ, US), without transfusion, a diaphragm lesion following by a pleural cavity opening, sutured without a pleural drainage and two splenic capsule lesions, conservatively and successfully treated. Conversion to open surgery was necessary in 1/88 patients (1.13%), due to suspected 8 cm PCC infiltration of the renal vessels, not confirmed at definitive histology, which reported a desmoplastic reaction. Mean hospital stay was 3.5 days (range 2-9).

**Table 2:** Perioperative results

Mean operative time (min)	137.33 (range 100-180)
**Intraoperative blood loss (ml)**	149.7 (range 50-280)

**PCC Hypertensive crises (SBP > 180 mmHg)**	4/17 (23.5%)

**PCC Hypotensive crises (SBP < 90 mmHg)**	2/17 (11.7%)

**Conversion to open procedure**	1/88 (1.13%)

**Morbidity **	5/88 (5.6%)

**Mean Hospital stay (days)**	3.5 (range 2-9)

PCC: Pheochromocytoma

#### Morbidity

No postoperative mayor complication occurred. 30-day morbidity rate was 5.7% (5/88 patients), and consisted of one case of abdominal wall hematoma, two cases of port site hernia, one case of pneumonia and one intra-abdominal collection spontaneously resolved. Pulmonary embolism, acute renal failure, bleeding requiring transfusion, deep venous thrombosis and sepsis were not observed during the 30 postoperative days.

There was no relationship between preoperative doxazosin dose and intra operative hypertensive crises, which occurred at induction of anesthesia and during manipulation of the adrenal gland in 4/17 patients (23.52%). At induction, 1/17 patients (5.88%) had paroxysmal hypertension with a peak of 220/110, resolved after a pharmacological treatment. Further episodes of hypertension, with a median BP of 230/140 mmHg (range 230/160 - 320/150 mm Hg), were observed during manipulation of the adrenal gland in 3/17 patients (17.64%), and ceased after adrenalectomy. The hypertensive crises were of short duration and were not associated with any significant complication such as cerebral vascular accident, pulmonary edema, myocardial infarction or ischemia, cardiac arrhythmias and multiorgan failure, and no postoperative mechanical ventilation was required. Regarding to the relationship between hypertensive crises and PCC size, 2 patients with PCC 3 and 8 cm in diameter had the highest BP levels (230/130-320/150 mmHg) (Figure 1). Specifically, the highest BP levels were observed during adrenal gland manipulation in a patient who underwent LA to remove a PCC 8 cm in diameter (320/150 mmHg). There was no statistical influence for anyone of the examined variable - age, size and operative time - on the occurrence of the intraoperative hypertensive crises. No intra operative hypotensive episodes were observed. After awakening from anesthesia, 1/17 patients (5.88%) was found to have hypotension (70/40 mmHg), which was treated by crystalloids and hydrocortisone. On the postoperative day 1, 1/17 patients (5.88%) had hypotension (80/40 mmHg) which was successfully treated by hydrocortisone and crystalloids. No relationship was observed between PCC operative times and neoplasms size ( =/< 6 cm or > 6 cm).

#### Follow-up

LA was efficacious in determining a normalization of the endocrine profile. One male patient, after resection of a PCC < 6 cm in diameter, underwent reoperation by open surgery via a posterior approach, due to disease persistence for remnant retrocaval adrenal tissue with persistent elevation of metanephrine levels. A Conn's male patient, about 8 years after a right LA, was submitted to a sparing ultrasound-assisted left LA for a metachronous contralateral tumor,. At 54.43 month follow-up (6-120 months), no complication and/or disease recurrence were observed. Two female patients, affected by breast and renal cancer, are alive respectively 2 and 4 years following LAs.

## Discussion

In the last 20 years, the wide diffusion of imaging determined an increase of adrenalectomies and nephrectomies [10,12,14]. Lower morbidity and mortality rates, decreased postoperative pain, analgesic administration, ileus and costs, shorter hospital stay, earlier return to work and a better cosmetic result than those reported following open surgery, are the main advantages of retroperitoneoscopic or laparoscopic adrenalectomy, largely established as the gold standard for small benign adrenal lesions. The complication rate ranges from 3 to 20% [12] and is similar to that reported in the management of other endocrine diseases [15-19]. Previous laparotomic surgery may be not considered as contraindication. Recovery from clinical syndromes associated with excessive hormonal secretion, is the major objective of surgery, but the large acceptance of the mini-invasive approach extended the indications to the treatment of incidentalomas > 3.5-4 cm, cysts, glandular hyperplasia and metastases [4,5,10,20]. Oncological outcomes of laparoscopic treatment of adrenal metastases were similar to those following traditional surgery [21].

In our experience, thanks to the improvement of the surgical skills and the technology assistance, i.e. new dissection instrumentations or intra operative ultrasound (very useful during a sparing adrenalectomy), LA was considered as the gold standard for the treatment of benign adrenal diseases, in such cases with a diameter up to 12 cm (myelolipoma) [7,20]. Surgical approach was effective, safe and well tolerated in most cases, with a very low morbidity rate, also following PCC treatment, considered at high risk for hemodynamic disorders [22]. A preoperative selective adrenergic blockade with doxazosin in PCC patients did not prevent intraoperative hyperthensive episodes, but major related cardiovascular complications - cerebral vascular accident, pulmonary edema, myocardial infarction or ischemia, cardiac arrhythmias and multiorgan failure - were not observed, although in some patients SBP rose up to 300 mmHg.

Authors analyzed their results throughout the discussion and performed a literature review. Although our study confirms previous published data, it has several limitations. Patients were operated by a single team, composed by experienced endocrine and laparoscopic surgeons, in a tertiary referral center, and no patient underwent a retroperitoneal approach. Finally, data were retrospectively evaluated and no comparison with an "open" series was carried out.

### Demographics

The presented series includes patients mostly affected by a clinical syndrome due to an excessive hormonal secretion, referred to our observation by endocrine referral units, presenting complex clinical pictures and needing often a preoperative medical treatment. In PCCs hypertension was paroxysmal or persistent, while 2/17 patients (11.76%) were normotensive. According to literature data, we recommend a pharmacological treatment by mean of doxazosin (2-10 mg daily) for at least 2 weeks preoperatively, even in normotensive patients [1,11,23-26]. In our series, the adrenergic blockade did not prevent intraoperative hypertensive episodes, but allowed their optimal pharmacological control: hypertensive crises were of short duration, were effectively treated and significant cardiovascular complications were not observed. Cushing's diseases were associated with cardiovascular disease, but nevertheless their morbidity rate was similar to that observed in patients affected by incidentalomas, that were less frequently observed in our series. In Conn's disease management, a contralateral metachronous aldosteronoma was reported, and, finally, the role of the sparing adrenalectomy should be carefully evaluated.

### Surgical details

We considered the first 30 operations as part of the learning curve, but about 40 cases are needed before mastering the surgical procedure. A higher risk of conversion to open surgery is identifiable in patients affected by adrenal lesions greater than 8 cm and needing concomitant surgical procedures, that should be not managed during the first phase of experience without a tutor [4]. Our low rate of conversion, similar during the learning curve and after this time, reflects the high proficiency in advanced laparoscopic surgery of the operative team, as well as confirming the appropriate indications to LA. According to literature guide lines [4-6,10,12,27,28] we operated on patients affected by PCC up to 8 cm, incidentalomas up to 6 cm and metastases < 6 cm. Nevertheless, tumor size should be not considered as the sole criterion in selecting the surgical approach, because large indeterminate mass or carcinoma and the need of concomitant procedures may significantly influence laparoscopic outcomes [4,29]. In case of suspected primary malignant adrenal tumor LA was contraindicated. Considering the poor surgical outcomes and the high risk of peritoneal dissemination during surgery, indications to LA for primary adrenal cancer are still a matter of debate [30-32]. Moreover, the risk of malignancy was considered in the past as a contraindication to the laparoscopic treatment of PCC >6 cm. Nevertheless, PCC >6 cm in diameter becomes malignant less frequently than other adrenal lesions of the same size do, and probably size cannot itself be an absolute contraindication, provided that there is no evidence of local invasion [6]. Adrenocortical carcinoma is more frequent in non PCC lesions >6 cm, that may be routinely considered as a contraindication to LA [30-32]. Even though family history, virilising features, mixed hormonal secretion, rapid enhancement and rapid washout on MRI contrast imaging are considered predictor factors of malignancy, nevertheless only local invasion or metastases are undoubted signs of adrenal cancer. A lateral position of about 30° is actually preferred in performing a right LA, as well as, during a left LA, a wide left colon mobilization is routinely carried out. Finally, an ultrasonic dissector of recent conception (Harmonic Ace^® ^Ethicon Endo-Surgery INC - Johnson & Johnson, NJ, USA) is considered as the preferred adrenal gland dissection instrument, and resulted very useful in maintaining a near bloodless field, as well as reported in the management of other endocrine diseases [33,34].

### Morbidity and outcomes

Large indeterminate adrenal neoplasm, carcinoma, a tumor size >6 cm, and the need for concomitant surgical procedures are considered the major variables predicting the 30-day morbidity [4]. According to our experience, adrenal metastases and PCCs resulted at higher risk of complications and conversion. Nevertheless, the morbidity rate was similar during the learning curve and after this time, and no patient was reoperated. In Cushing's diseases, a higher post-operative infectious complication rate was not observed. In no patient pancreatitis or torsion of splenic vessels were observed. Considering a case of contralateral metachronous aldosteronoma relapse in a Conn's disease case, the role of ultrasound assisted sparing adrenalectomy should be carefully considered, especially in young patients. However, its indication remains controversial. In case of intraoperative complications, a conversion to open surgery was not necessary, and a "conservative" management of inferior vena cava, diaphragm, splenic and hepatic capsule injuries was easily performed, without excessive risks for the patients.

### Learning points

LA is safe and feasible also for large adrenal lesions and an additional port may be very useful, especially in case of complications. PCC surgery may be associated with higher operative time, complications and conversion rate, and an experienced operative team is needed. Previous laparotomic surgery may be not considered as contraindication to a laparoscopic approach, and moreover, the surgical skill required is proportional to the tumor size. A collaborative management, between endocrine physician, endocrine surgery and anesthesiologist is recommended in referral high volume units, especially in the treatment of PCC or Cushing's disease patients, considered at high risk of morbidity. To enhance the surgical performance, the operative team should be composed by individuals together performing a LA repetitively. Major intraoperative complications may be laparoscopically managed, avoiding a laparotomy, only if great care is adopted, and a prompt conversion is considered in case of technical difficulties, to reduce patient risks. Preoperative αblockade does not prevent PCC hyperthensive crises, but, facilitating their pharmacological control, must be recommended. A genetic test for RET, Von Hippel-Lindau (VHL), Neurofibromatosis type 1 (NF1), and of the genes coding for the different subunits of the succinate dehydrogenase (hereditary paraganglioma syndromes) is suggested in familial PCC to obtain a precocious diagnosis.

## Abbreviations

LA: laparoscopic adrenalectomy; PCC: pheochromocytoma; MA: minimally invasive adrenalectomy; ASA: American Society of Anestesiology; BP: blood pressure; CT: computed tomography; SBP: systolic blood pressure; HR: Heart rate; MEN: multiendocrine neoplasia; MRI: Magnetic resonance imaging; VHL Von Hipple Lindau; NF1: neurofibromatosis type 1.

## Competing interests

The authors declare that they have no competing interests.

## Authors' contributions

GC: conception, design, and execution of the study; analysis and interpretation of data; drafting and editing of the manuscript. DP: conception and design of the study; analysis and interpretation of data. CDP: conception, design, and execution of the study; analysis and interpretation of data. SN: drafting and editing of the manuscript. DE: conception, design, and execution of the study. SI: conception, design, and execution of the study. ADB: conception and design of the study; analysis and interpretation of data. GD: conception and design of the study; analysis and interpretation of data. FF: conception, design, and execution of the study; drafting and editing of the manuscript. LS: conception and design of the study; analysis and interpretation of data. AAS: conception and design of the study; analysis and interpretation of data.

## Authors' information

GC: Assistant Professor of Surgery

DP: Assistant Professor of Endocrinology

CDP: Surgical Fellow

SN: Surgical Fellow

DE: Surgical Fellow

SI: Medical Doctor

ADB: Associate Professor of Endocrinology

GD: Associate Professor of Surgery

FF: Assistant Professor of Anaesthesiology

LS: Full Professor of Surgery

AAS: Associate Professor of Endocrinology

## Declaration statement

Publication of this article was funded by personal funds.

This article has been published as part of *BMC Surgery *Volume 13 Supplement 2, 2013: Proceedings from the 26th National Congress of the Italian Society of Geriatric Surgery. The full contents of the supplement are available online at http://www.biomedcentral.com/bmcsurg/supplements/13/S2
